# Sudden perioperative death post aortic valve replacement with autopsy showing hypertrophic cardiomyopathy in elderly female

**DOI:** 10.1007/s00414-025-03544-9

**Published:** 2025-10-02

**Authors:** Zubair Abdul Razak, Joseph Westaby, Mary N. Sheppard

**Affiliations:** 1 Department of Forensic Medicine, Hospital Sultan Ismail, Johor Bahru, Malaysia; 2 CRY Cardiovascular Pathology Department, St George’s Hospital Medical School, Cardiovascular and Genetic Research Institute, University of London, London, UK

**Keywords:** Aortic valve replacement, Aortic valve stenosis, Hypertrophic cardiomyopathy, Postoperative death

## Abstract

**Introduction:**

Perioperative death is a catastrophic event for the family and the surgical team. It is in the interest of both that an autopsy is carried out to explain the death. We report a sudden perioperative death post aortic valve replacement in an undiagnosed hypertrophic cardiomyopathy (HCM) in an elderly female.

**Case report:**

A 73-year-old female underwent an elective aortic valve replacement (AVR) due to severe aortic valve stenosis (AS). The operation went smoothly. However, left ventricular function was poor despite maximal inotropic treatment. The left ventricular function never recovered and she died on the operating table. The heart weight was normal but the left ventricle shows septal hypertrophy (20 mm). Histology of the left ventricle showed florid myocyte disarray indicating hypertrophic cardiomyopathy.

**Discussion:**

AS is the most common Valvular Heart Disease, and most patients undergo AVR. Nevertheless, sudden unexpected death remains a common cause of late mortality after successful valve replacement. Surprisingly this lady died just after the operative procedure. Histological examination confirmed HCM. HCM is an inherited cardiac condition and it is important for the family to be screened to prevent future sudden cardiac death.

**Conclusion:**

This case highlights the importance of autopsy in a post-operative death case. It can be of great value to the surgical team and family members.

**Supplementary Information:**

The online version contains supplementary material available at 10.1007/s00414-025-03544-9.

## Introduction

Perioperative and Post-operative death is a catastrophic event not just for the family but also the surgical team. It is in the interest of both that an autopsy is carried out to explain the death. We report a sudden perioperative cardiac death post aortic valve replacement (AVR) in which the heart showed undiagnosed hypertrophic cardiomyopathy (HCM) in an elderly female explaining the catastrophic perioperative cardiac arrest. This explains the death to the family and also has major genetic implications for other family members.

## Case report

A 73-year-old female underwent an elective, low risk AVR due to severe aortic valve stenosis (AS). The operation went smoothly with no technical issues. The aortic valve prosthesis was well seated. However, left ventricular function was poor despite maximal inotropic treatment. The surgeons did two left-sided vein bypasses for left anterior descending and circumflex artery to ensure adequate blood supply to the left ventricle. Despite this procedure the left ventricular function never recovered and she died on the operating table. An autopsy was ordered by the local coroner.

**Fig. 1:**
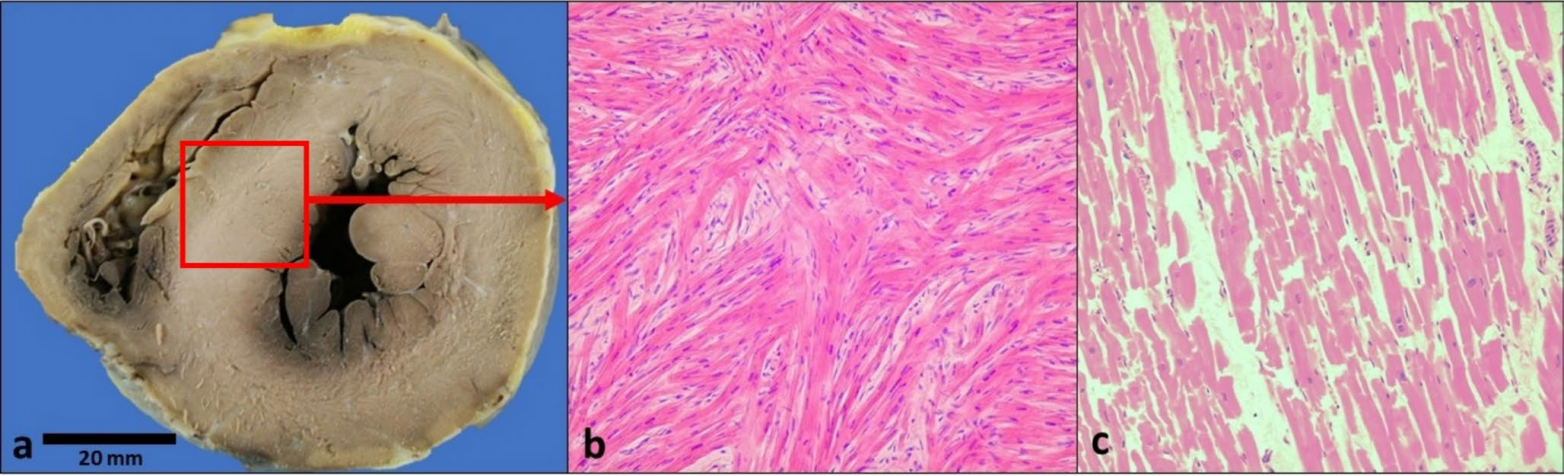
**(a-c)**. **Figure a**: Mid-ventricular slice of heart is showing septal hypertrophy of 20 mm. **Figure b**: Red arrow points to mid septal sampling for histology which demonstrate myocyte hypertrophy and widespread diffuse individual and bundle myocyte disarray with cells showing bizarre shapes and size with myocytes diverging from normal parallel pattern around capillaries. **Figure c**: Illustrate normal parallel pattern of myocytes found in normal heart

Examination of the heart showed normal size weighing 380 g. There were two vein grafts to the left anterior descending and circumflex artery. The left ventricle did show septal hypertrophy with a thickness of 20 mm with no macroscopic scarring (Fig. 1a). There was no impact lesion. Aortic valve was replaced by a trileaflet bioprosthetic valve which had no complications. Other valves were normal. All coronary arteries were normal with no significant atheromatous narrowing.

Microscopic examination of 5 sections of the left ventricle showed myocyte hypertrophy with nuclear pleomorphism and hyperchromasia. There was widespread diffuse individual and bundle myocyte disarray present throughout the septum and free wall of the left ventricular with increased interstitial fibrosis (Fig. 1b). The intramural vessels showed smooth muscle hyperplasia [5].

This is a death occurring following surgery for aortic stenosis with a bioprosthetic AVR in which the heart is of a normal weight (380 g) but showed myocyte hypertrophy with nuclear pleomorphism and hyperchromasia, with widespread diffuse individual and bundle myocyte disarray throughout the left ventricular wall indicating HCM at autopsy. Widespread diffuse individual and bundle myocyte disarray is not seen in isolated aortic stenosis. This is responsible for the post-operative death with poor left ventricular function.

## Discussion

AS is the most common Valvular Heart Disease (VHD) in the western world; >400,000 people underwent AVR in the USA alone in 2019 [1]. AVR has become the standard therapy for severe aortic valve disease which usually results in major clinical improvement. However sudden unexpected death remains a common cause of late mortality after successful valve replacement.

In a study of 599 patients, 41 died within 4 weeks postoperatively, resulting in an overall perioperative mortality of 6.9%. During the mean observation period of 4.7 years (1–14 years), an additional 83 patients died, with late annual mortality of 3.6%. The deaths were sudden in 20 patients (24%) [2]. Our patient died just after the operative procedure when the left ventricular function could not be restored despite maximum support and two vein grafts bypasses. Clinically there was no history of HCM in this patient and she had a normal heart weight at autopsy. It took histological examination of the heart to give the answer and this diagnosis was important for the family and the surgical team in order to come to terms with the death with cardiological screening of the family being advised.

Sheppard et al. [5] stated that in HCM, macroscopically, the heart is usually enlarged but it can be normal weight. Septum and LVwall thickness are usually increased more than 15 mm. The anterior leaflet of the mitral valve can be thickened with an impact lesion. The majority have an asymmetrical pattern of hypertrophy. Microscopically, myocyte disarray is the most important diagnostic histological feature of HCM. Cardiac myocytes show hypertrophy with bizarre myocytes shapes (disarray), nuclear pleomorphism and hyperchromasia. Interstitial fibrosis is often as present. Intramural vessels show thickened walls with increased intimal and medial collagen and narrowed lumen. Most of these features were seen in this case except for thickened anterior leaflet of the mitral valve and impact lesion.

HCM is a familial genetic disorder which is due to mutation in sarcomere proteins, although several other disorders have also been linked to the HCM phenotype with a prevalence of 1: 500 in a general population of healthy young adults [3]. The condition is autosomal dominant and it is recommended for relatives to be screened by a cardiologist with an interest in inherited cardiac condition. SCD is often the first manifestation of HCM and a normal heart weight can be found in up to 30% of cases [3, 4].

## Conclusion

This case highlights the importance of autopsy in a post-operative death case. It can be of great value to the surgical team and family members. This autopsy revealed underlying HCM which is a genetic heart disease and was responsible for the death of the patient and has significant implications for the family. The family was informed about the cause of death and was referred to a cardiologist for inherited cardiac condition screening with future genetic testing of family members. Histology is crucial in the diagnose HCM because it can present even in a normal weight heart as in this case Forensic pathologists should be aware that a pathological diagnosis of HCM is made by the detection of significant myocyte disarray and not by genetic testing [5]. This is the first report of such a case in the literature to our knowledge.

## Electronic supplementary material

Below is the link to the electronic supplementary material.

Supplementary Material 1
