# Supplementary material for: Sudden perioperative death post aortic valve replacement with autopsy showing hypertrophic cardiomyopathy in elderly female
Source: Int J Legal Med. 2025 Oct 2;140(1):249–51. doi: 10.1007/s00414-025-03544-9 (PMC12808209; doi:10.1007/s00414-025-03544-9)
Supplement: Supplementary file 1 — Supplementary Material 1 [file 414_2025_3544_MOESM1_ESM.docx]

**Author Contributions Declaration**

All authors contributed to the manuscript. The first draft of the manuscript was written by Zubair Abdul Razak. Joseph Westaby and Mary N. Sheppard commented on previous versions of the manuscript. All authors read and approved the final manuscript.
